# Association between social support and post-traumatic stress disorder symptoms among Chinese patients with ovarian cancer: A multiple mediation model

**DOI:** 10.1371/journal.pone.0177055

**Published:** 2017-05-05

**Authors:** Chunli Liu, Yi Zhang, Hong Jiang, Hui Wu

**Affiliations:** 1 Library of China Medical University, Shenyang, Liaoning, China; 2 Department of Social Medicine, College of Public Health, China Medical University, Shenyang, Liaoning, China; 3 The First Affiliated Hospital of China Medical University, Heping District, Shenyang, Liaoning, China; 4 School of Public Health, China Medical University, Shenyang, Liaoning, China; National Cancer Center, JAPAN

## Abstract

Post-traumatic stress disorder (PTSD) symptoms can develop after person experiences one or more traumatic events. Little research, however, has been done on PTSD symptoms of patients with ovarian cancer. The present study aimed to estimate the prevalence of PTSD symptoms in patients with ovarian cancer in China; the effects of demographic and clinical variables on PTSD symptoms; multiple mediation roles in the association between social support and PTSD symptoms in patients with ovarian cancer in China. We collected demographic and clinical information of patients with ovarian cancer in the first and second hospitals of China Medical University between January 1, 2014 and December 31, 2015. Qualified patients were asked to complete the Posttraumatic Stress Disorder Checklist-Civilian Version (PCL-C), Duke-UNC Functional Social Support Questionnaire, Herth Hope Index (HHI), and Resilience Scale-14 (RS-14). 201 patients provided responses. We performed hierarchical linear regression to assess the correlation between social support and PTSD symptoms and bootstrapping to test the mediating role of hope and resilience as potential mediators. After controlling demographic and clinical characteristics, social support negatively correlated with PTSD symptoms (β = -0.406, P < 0.01). Social support explained 14.7% of the variance in PTSD symptoms. Hope and resilience explained 17.0% of the variance in PTSD symptoms. The proportion of the hope mediating effect was 43.37% for social support and the proportion of the resilience mediating effect was 10.64% for social support. Hope and resilience partly mediated the correlation between social support and PTSD symptoms despite accounting for different proportions of the mediating effect. Future intervention plans should pay more attention to social support as well as hope and resilience to prevent, relieve and treat PTSD symptoms.

## Introduction

Ovarian cancer is a gynecological malignancy disease that causes more deaths than other female reproductive cancers. Most patients are diagnosed ovarian cancer at a late stage, and the prognosis is poor [1–3]. The incidence rate and survival rate vary by geographical region and population. According to epidemiology statistics in UK, ovarian cancer is the sixth most common cancer in women [4], while it is the greatest cause of gynecological cancer death, with a 46% 5-year survival rate. In the US [5], while it is the eighth most common cancer among women, ovarian cancer is the fifth leading cause of cancer-related death among women, and is the deadliest of gynecologic cancers, with a 45.6% 5-year survival rate. In China, ovarian cancer is the second most common cancer in women behind cervical cancer, and the mortality rate is 21.6%, ranking as the deadliest cancer among women [6].

Cancer and its treatment can bring significant physical and psychological trauma. Patients and even their partners are continuously bearing the fear of death as well as pain, stress, and the cost [7–10]. Besides the economic burden and mental pressure, patients with ovarian cancer have to suffer terrible pain from surgery and side effects of chemical and radiation therapy. Approximately one fifth of women self-reported different degrees of distress (more than half with high psychological stress or mental disorder) due to the diagnosis of ovarian cancer and corresponding treatment [11].

Post-traumatic stress disorder (PTSD) is the appearance of delayed issues and continued mental disturbance after an individual experiences, witnesses, or encounters one or more deaths of someone related to themselves or others, or a threat of death or serious injury, according to the Fifth Edition of the Diagnostic and Statistical Manual of Mental Disorders (DSM-5). The diagnostic criteria of PTSD emphasize direct exposure, witnessing in person, learning that a close relative or close friend experienced trauma, or repeated indirect exposure to adverse events. PTSD includes three types of symptoms: hyper vigilance (always being on alert, trouble sleeping, irritability, difficulty concentrating), avoidance of stimuli related to the traumatic event (such as persons, places, or situations) and intrusive thoughts (re-experiencing the trauma, such as nightmares, extreme distress, frequent and distressing memories). PTSD symptoms can also result in job loss, substance abuse, and other stressful problems [12].

Some researchers have found a high prevalence of PTSD-related symptoms in patients who have suffered from newly diagnosed cancer or cancer resection [13–17]. Among women, a few studies have focused on prevalence and risk factors of PTSD symptoms in patients with breast cancer [18–21] and ovarian cancer [22–23]. For example, there is evidence of a decline in PTSD symptoms rates over time in a sample of 121 UK outpatients with ovarian cancer [22]. Another study surveyed 108 Australian women with ovarian cancer, and found that 9.25% suffered from PTSD symptoms, 5.6% from depression, and 13.9% from anxiety [23]. However, there is little study concerning the prevalence of PTSD symptoms among ovarian cancer patients in China. In addition, PTSD symptoms vary according to a series of variables: time since diagnosis or treatment, type of treatment, income, marital status, cancer stage, and treatment type [24–25]. Similarly, little evidence concerning which demographic and clinical characteristic of patients significantly show impact on the PTSD symptoms among ovarian cancer patients in China has been demonstrated.

Social support refers not only to material support but also to spiritual help that actually or apparently comes from the community, social network, and other people who can be trusted. Social support primarily covers emotional support, social integration or network support, support to improve self-esteem, financial support, information support, and accompanying support, and so on. Social support [26–27] has been found to promote mental adjustment in conditions with chronic high stress like cancer. People with higher levels of social support may adjust to crises more smoothly [28]. On the contrary, people with lower social support may have more difficulty recovering from trauma.

Does social support or other positive psychological factors show effect on PTSD symptoms after cancer? Recently, social support has shown negative correlations with PTSD symptoms [29–30]. Having a good social support system can prevent you from PTSD symptoms. Dinenberg RE[31] concluded that social support was strongly associated with future PTSD symptoms in patients with cardiovascular disease. They indicated that to help patients from risk of developing PTSD symptoms, optimize the social support level might be a good intervention method. In turn, PTSD symptoms may affect social support. A study on United States veterans from Afghanistan/Iraq era suggested that veterans with PTSD symptoms might have difficulty in accepting or benefitting from social support[32]. Moreover, some studies indicated that social support correlates with recovery from PTSD symptoms [33–34]. Given the collectivist culture in China, social support may be relevant to the outcomes in that social support is particularly important to Chinese women. However, little work has investigated the impact of social support on PTSD symptoms in Chinese patients with ovarian cancer.

Furthermore, if social support and PTSD symptoms are related, hope and resilience may explain or mediate this relationship. Resilience is an individual characteristic of power or quality. For example, psychological resilience, which serves as an ability to adapt to the changing environment flexibly, can accelerate recovery from negative experiences [35–36]. Resilience can give individuals psychological strength to solve a series of problems after disaster or trauma. Resilient individuals are able to utilize their skills and strengths to recover from stress or challenges. However, little evidence has documented the complex relationship between resilience and PTSD symptoms in patients with cancer, especially in ovarian survivors [37]. Hope is also an important impact factor for the lives of cancer patients. Miller defined hope as anticipation of a continued good state or an improved state [38]. To the ill populations, hope is anticipation for a good future, based on the ability of coping with illness, the relationships with others, psychological well-being, and others [38]. What is more, hope means not only the sense of hope, but also the desire for interpersonal relationships, even engagement in the relationships, and the controlling of emotional responses[39]. He developed a model of hope with three dimensions: inner sense of temporality and future (such as presence of goals; scared about the future); positive readiness and expectancy (for example, a sense of direction; life has value and worth); interconnectedness (for instance, give and receive caring/love) [40]. To the cancer patients, continuous negative mood will affect physical and mental state. Many cancer patients have to struggle with the PTSD symptoms including insomnia, nightmares, anxiety and hyper-vigilance and even enormous despair [41–43]. In this case, hope is very essential to the adaption to illness and the recovery from the PTSD symptoms.

Then, is there any evidence that social support correlates with hope or resilience? Quite a few years ago, some researchers have studied this question and provided evidence that social support promote the resilience and hope. Jennifer L. Mattioli[44] has conducted semi-structured interviews to explore and describe what hope and social support mean to the patients who are receiving chemotherapy. Social support from family, friends, and healthcare providers is helpful for patients to deal with their disease treatment. Hope aids in people overall health and well-being. Therefore, it is understandable that people with good social support keep more optimistic toward their life, and then become more active and hopeful to conquer or coexist with cancer. In a word, there may be direct relationship between social support and hope. When social support increases, hope will elevate accordingly[45–46].

Lauren M. Sippel [47] has studied the correlation between social support and individual resilience. The findings suggest that high social support can increase self-confidence, foster more active problem solving, and then elevate individual resilience in the capacity to adapt well in the face of adversity. Also, findings[48] from laboratory-based studies in which participants undergo cardiovascular monitoring have provided evidence that social support can nourish further emotional resilience, or in other word, social support may be effective in elevating resilience. Phoenix Mo [49] examined the relationship between social supports, resilience, hopelessness, posttraumatic growth among children of HIV-infected parents in China. Results suggested that social support had a significant positive relationship with resilience and Posttraumatic growth. Wilks SE found that social support from family positively influenced resilience in Alzheimer’s disease caregivers[50].

In this perspective, we posit that in our sample of ovarian cancer patients, social support show negative effect on PTSD symptoms and positive effect on both hope and resilience. Besides that, hope and resilience may mediate the relation between social support and PTSD symptoms. The aims of the present study were to estimate: (1) the prevalence of PTSD in patients with ovarian cancer in China; and (2) the effects of demographic and clinical variables on PTSD symptoms; and (3)a multiple mediation in the association between social support and PTSD symptoms in patients with ovarian cancer in China.

## Methods

### Ethics statement

The Committee for Human Trials of the China Medical University revised and approved the research and study procedures were in accordance with the ethical standards. All patients gave their permission to participate after being verbally informed of the study protocol. Participation was completely voluntary and anonymous. We protected the privacy of personal data processing and maintained confidentiality of individual records and accounts. Participation in this study did not affect future free medical examination and treatment that is standard in China.

### Study design and sample

From January 1, 2014 to December 31, 2015, we used convenience sampling to recruit patients with ovarian cancer in the first and second hospitals of China Medical University, which are important providers of gynecologic surgery in the northeastern region of China. Patients who satisfied the following inclusion criteria were enrolled as potential subjects: (1) at least 18 years old when diagnosed with ovarian cancer, (2) histological evidence of ovarian cancer, (3) able to speak and read Chinese and fill in the questionnaire, and (4) clear consciousness and cognition (can correctly answer place and characters within 30 seconds). Exclusion criteria were as follows: (1) those with psychiatric problems such as depression, anxiety, or other psychiatric disorders before being diagnosed with cancer, (2) those with intellectual abnormalities, and (3) patients who had other types of cancers. After obtaining written informed consent from patients, clinical data were collected from their medical records and a structured questionnaire was distributed. A total of 220 patients with ovarian cancer were enrolled. Five patients refused to join the survey. Of the 215 eligible patients, 14 were excluded from analysis (more than 30% missing data). Finally, we received effective responses from 201 patients (effective response rate of 93.5%).

### Demographic and clinical characteristics

We obtained four demographic characteristics including age, education and marital status. Educational level was divided into primary school, secondary school, and junior college or above. Income was divided into≤2000, 2001–3000and>3000. Three clinical characteristics obtained were cancer stage, treatment type, and metastasis. Cancer stage was dividedintoI, II, III, andIV. Treatment type was dividedinto no treatment, chemotherapy, surgery, and combined treatment. Metastasis was divided into no and yes.

### Post-traumatic stress disorder symptoms

We used the PTSD Checklist-Civilian Version (PCL-C) [51], a 17-item questionnaire to assess the degree of PTSD symptoms. Each item was rated on a 5-point Likert scale (1 = not at all; 5 = extremely) based on how much the symptom was bothersome in the last month. Total scores range from 17 to 85. Scores equal to or greater than 44 are considered to indicate PTSD symptoms[52]. In this study, patients were asked to comment on items of the PCL-C in their experience with ovarian cancer and its treatment. The Chinese version of the PCL-C has been used in the Chinese population, demonstrating sufficient reliability [53–54]. In our study, Cronbach’s alpha for the total scale was 0.940.

### Social support

We chose the Duke-UNC Functional Social Support Questionnaire [55] to assess social support. This is an 8-item questionnaire, where each item is rated on a 5-point Likert scale (1 = much less than I would like; 5 = as much as I would like) based on satisfaction with perceived social support. Studies using the Duke-UNC Functional Social Support in Chinese populations have shown that it has good reliability [56]. In our study, Cronbach’s alpha for the total scale was 0.886.

### Hope

We selected the Herth Hope Index (HHI) [57], which is an adaptation of Herth Hope Scale (HHS) to assess hope of patients. It is a questionnaire including 12 items, and each item is rated on a 4-point Likert scale (1 = strongly disagree; 4 = strongly agree). The total score ranges from 12 to 48. The Chinese version of the HHI has been used in Chinese patients with cancer, and demonstrated satisfactory reliability and internal consistency [58]. In this study, Cronbach’s alpha for the total scale was 0.840.

### Resilience

We used the Resilience Scale-14 (RS-14) [59] to test the resilience level of patients. The RS-14 is a 14-item questionnaire, where each item is rated on a 4-point Likert scale (1 = entirely not match; 4 = completely match). The total score ranges from 14 to 56. Some studies have verified the reliability of the Chinese version [60]. In our study, the Cronbach’s alpha value for the total scale was 0.906.

### Statistical analysis

We used SPSS 17.0 for Windows to perform all analyses. All statistical tests were two-sided (α = 0.05). Descriptive statistics for demographic and clinical variables are shown with mean, standard deviation (SD), number (N), and percentage (%), as appropriate. Variation in PTSD symptoms was examined with regard to demographic and clinical variables using independent samples t-tests and one-way analyses of variance (ANOVAs). Correlations among PTSD symptoms, social support, hope, and resilience were preliminarily examined by Pearson’s correlation analysis.

We performed hierarchical multiple regression analysis to explore the effects of groups of independent variables on PTSD symptoms. There were three blocks of independent variables. In block 1, all demographic and clinical variables were entered as control variables. Because marital status, educational level, income, cancer stage, treatment type, and metastasis are categorical variables without a linear trend, we set dummy variables for them [61]. In block 2, social support was added. Hope and resilience were added in block 3.

**Fig 1:**
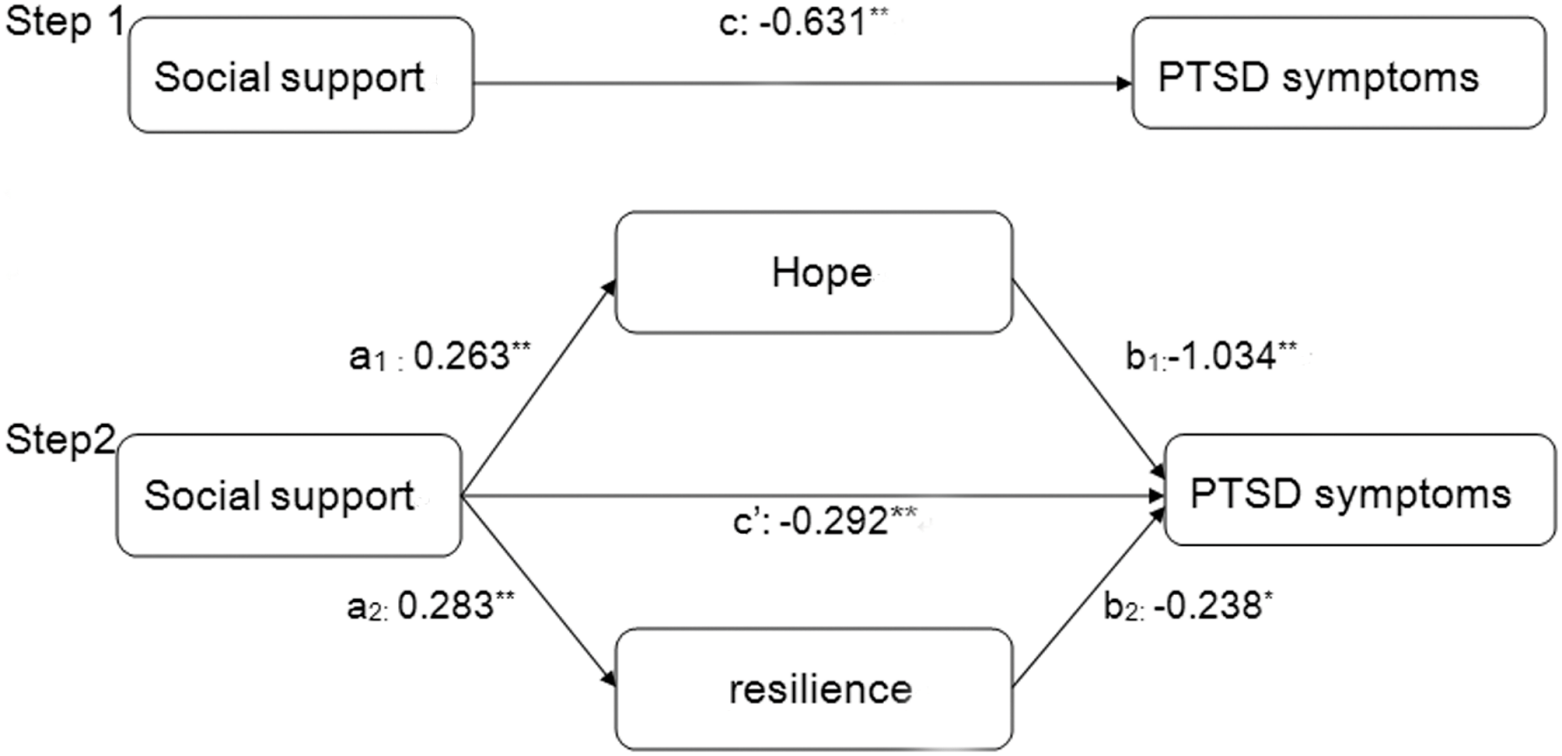
Theoretical model of the mediating role of hope and resilience on the relationship between social support and PTSD symptoms. (c) The association between social support and PTSD symptoms; (a_1_) The relationship between social support and hope; (b_1_) the relationship of hope with post-traumatic stress disorder (PTSD) symptoms after controlling the independent variables;(a_2_) The relationship between social support and resilience; (b_2_) the relationship of resilience with post-traumatic stress disorder (PTSD) symptoms after controlling the independent variables; (c’) the association between social support and PTSD symptoms after adding hope and resilience as mediators.

We used asymptotic and resampling strategies to examine the mediating roles of hope and resilience on the association between social supports on PTSD symptoms [62]. Social support was modeled as an independent variable, with PTSD symptoms as the dependent variable, and hope and resilience as mediators (as shown in Fig 1). Age, income and cancer stage were considered as covariates. In the first step, the aim is to identify the correlation between social support and PTSD symptoms (the c path). In the second step, the aim is to examine the mediation of hope and resilience (the a × b path). Standards we used to judge the mediating role are the same as we have used in previous studies [63]. We used bias-corrected and accelerated 95% confidence intervals (BCa 95% CIs). If zero is not included in the BCa 95% CI, then the mediation role (c’) is statistically significant. In the present study, we used 5000 bootstrap samples.

Fig 1 depicts a multiple mediation model with two mediators. Step 1 represents the total effect of social support on PTSD symptoms (path c). Step 2 represents both the direct effect of social support on PTSD symptoms (path c’) and the indirect effects of social support on PTSD symptoms via mediator hope and resilience. The specific indirect effectis the product of a and b. Hence, the total indirect effect can be calculated by the equation: c-c’ = a_1_×b_1_+a_2_×b_2_

## Results

### Demographic and clinical statistics and prevalence of PTSD symptoms

Demographic and clinical characteristics of the participants are shown in Table 1. Of the 201 respondents, age ranged from 24 to 79, and the average age was 55.28 ± 9.65years. Approximately 91.0% of respondents were married or living with a partner, and 59.7% received secondary school education. Nearly one half of respondents had a household income equal to or less than 2000. Of all respondents, 72.6% were diagnosed at III and IV cancer stage, and 57.2% received combined treatment. Metastasis was not present in 93.0% of respondents. Based on the cut-off values recommended byBlanchard, E. B. (≥44), the prevalence of PTSD symptomsamong ovarian cancer patients in Chinawas 21.9%.

**Table 1:** Demographic and clinical characteristics (N = 201).

Variable	N (%)	PTSD symptoms(Mean ± SD)	P-value
Demographic variables			
Age (years)			0.474
≤45	22 (10.9)	32.82 ± 10.82	
46–55	82 (40.8)	35.23 ± 11.09	
≥ 56	97 (48.3)	35.99 ± 10.99	
Marital status			0.263
Married/living with a partner	183 (91.0)	35.06 ± 10.85	
Single/widowed/divorced	18 (9.0)	38.11 ± 12.47	
Educational level			0.137
Primary school	54 (26.8)	37.28 ± 9.33	
Secondary school	120 (59.7)	35.18 ± 11.87	
Junior college or above	27 (13.4)	32.15 ± 9.51	
Income (yuan per month)			0.024
≤ 2000	98 (48.7)	37.27 ± 10.80	
2001–3000	61 (30.3)	34.59 ± 11.99	
> 3000	42 (20.8)	31.90 ± 9.05	
Clinical variables			
Cancer stage			0.010
I	36 (17.9)	30.69 ± 8.96	
II	19 (9.5)	33.58 ± 7.38	
III and IV	146 (72.6)	36.71 ± 11.54	
Treatment type			0.864
No treatment	13 (6.5)	36.62 ± 13.96	
Chemotherapy	60 (29.8)	35.55 ± 13.11	
Surgery	13 (6.5)	33.08 ± 5.12	
Combined treatment	115 (57.2)	35.33 ± 9.96	
Metastasis			0.299
No	187 (93.0)	35.11 ± 11.24	
Yes	14 (7.0)	38.29 ± 6.72	

### Effects of demographic and clinical variables on PTSD symptoms

As shown in Table 1, respondents whose income was equal to or less than 2000 had a high level of PTSD symptoms (37.27 ± 10.80), as compared to respondents with income in the 2001–3000 range (34.59 ± 11.99) or higher than 3000 (31.90 ± 9.05). In addition, respondents whose cancer stage was III and IV had a high level of PTSD symptoms(36.71 ± 11.54), as compared to stages I (30.69 ± 8.96) and II (33.58 ± 7.38).

### Pearson’s correlations among PTSD symptoms, social support, hope, and resilience

Pearson’s correlation coefficients were calculated between social support, hope, resilience, and PTSD symptoms. As shown in Table 2, PTSD symptoms were negatively associated with the three positive psychological variables (social support: r = -0.41, P < 0.01; hope: r = -0.54, P < 0.01; resilience: r = -0.36, P < 0.01). Social support was positively related with hope (r = 0.46, P < 0.01) and resilience (r = 0.25, P < 0.01). Moreover, hope was positively correlated with resilience (r = 0.39, P < 0.01).

**Table 2:** Means, SDs, and correlations among study variables.

Variables	Mean ± SD	Social support	Hope	Resilience	PTSD symptoms
Social Support	28.98 ± 6.88	1	0.46**	0.25**	-0.41**
Hope	35.40 ± 4.11		1	0.39**	-0.54**
Resilience	37.29 ± 7.28			1	-0.36**
PTSD symptoms	35.33 ± 11.00				1

**P< 0.01.

### Association between social support and PTSD symptoms by hierarchical linear regression

Table 3 shows the results of the hierarchical regression analysis. After controlling for age, income, and cancer stage, social support was negatively associated with PTSD symptoms (β = -0.406, P < 0.01) and explained 14.7% of the variance in PTSD symptoms. While hope and resilience were negatively associated with PTSD symptoms (β = -0.459, P < 0.01; β = -0.135, P < 0.05) and explained 17.0% of the variance in PTSD symptoms. In addition, after adding hope and resilience in the regression model of PTSD symptoms, the regression coefficient (absolute value of regression coefficient when it is negative) for social support reduced(from β = -0.406 to β = -0.136). Hence, we preliminarily consider that hope and resilience might play a mediating role between social support and PTSD symptoms. However, this point still needs further test by bootstrapping.

**Table 3:** Hierarchical linear regression for exploring the associated variables of PTSD symptoms.

Variables	PTSD symptoms		
	Step 1 (β)	Step 2 (β)	Step 3 (β)
**Block 1**			
Age	0.052	0.088	0.015
Income 1	0.196	0.130	0.046
Income 2	0.253*	0.241*	0.175
Cancer stage 1	0.063	0.075	0.024
Cancer stage 2	0.223*	0.162*	0.158*
**Block 2**			
Social support		-0.406**	-0.136
**Block 3**			
Hope			-0.459**
Resilience			-0.135*
F	1.779	4.769**	8.891**
Adjusted R^2^	0.045	0.197	0.372
ΔR^2^	0.102	0.147**	0.170**

*P< 0.05,

**P< 0.01.

Income 1 means 2001–3000 vs. > 3000; Income 2 means ≤2000 vs.>3000;

Cancer stage 1 means II vs. I; Cancer stage 2 means III and IV vs. I.

### Bootstrapping test of the mediating role of hope and resilience

Path coefficients a (between social support and mediators) and b (between mediators and PTSD symptoms), a×b products are presented in Table 4. Social support was significantly and positively associated with hope and resilience. Consistent with the results from hierarchical multiple regression analysis, hope and resilience were significantly and negatively associated with PTSD symptoms after controlling for age, income, cancer stage and social support. Each BCa 95% CI for a × b of hope and resilience excluding 0 indicated their significant mediation when they are added in the model. Thus, significant mediating roles of hope (a×b = -0.274, BCa 95% CI: -0.405, -0.158), and resilience(a×b = -0.067, BCa 95% CI: -0.145, -0.008) on the association between social support and PTSD symptoms were revealed among patients with ovarian cancer in China.

**Table 4:** Bootstrapping test of the indirect effect of hope and resilience on the relation between social support and PTSD symptoms.

(N = 201)	Mediators	c	a	b	c’	a × b (BCa 95% CI)
PTSD symptoms	Hope	-0.631**	0.263**	-1.034**	-0.292**	-0.274 (-0.405, -0.158)
	Resilience	-0.631**	0.283**	-0.238*	-0.292**	-0.067 (-0.145, -0.008)

Notes: c: correlation of social support with PTSD symptoms; a_1_: correlation of social support with hope; b_1_: correlation of hope with PTSD symptoms after controlling for the predictor variables; a_2_: correlation of social support with resilience; b_2_: correlation of resilience with PTSD symptoms after controlling for the predictor variables; c’: the association between social support and PTSD symptoms after adding hope and resilience as two mediators; a ×b: the product of a and b; BCa 95% CI: the bias-corrected and accelerated 95% confidence interval; age, income and cancer stage were covariates;

* p<0.05,

** p<0.01.

We used the formula (a × b / c) to calculate the proportion of mediation role. The proportion of the mediating effect accounted for by hope was 43.37% for social support, and that of resilience was 10.64%. Hence, the total effect of social support on PTSD symptoms by two mediators’ role (from hope and resilience together) was 54.00%.

## Discussion

We used self-report checklist to measure the prevalence of PTSD symptoms among patients with ovarian cancer in China. The prevalence of PTSD symptoms was 21.89%, which is higher than that among Chinese individuals with hematological malignancies(10.7%) [64], Australian women with ovarian cancer (9.25%) [23], and lower than that among Chinese women with bladder and kidney cancer (25.2%) [65]. The prevalence of PTSD symptoms correlates with the PTSD checklist version[66] and the cut-off score that is used [67–68]. In addition, the comparison of the prevalence of PTSD would be challenging without considering the different study procedures and populations across studies [69].

Demographic and clinical characteristics also affect the level of PTSD symptoms. Income and cancer stage are two significant factors that affect PTSD symptoms. Given the significant economic pressure in the treatment of ovarian cancer, the psychological burden of patients with a lower income (less than 2000) was heavier than it was for those with a higher income (more than 3000). It is indicated from our results that the level of PTSD symptoms in stages III and IV was higher than those in stage I. Thus, in psychological counseling and clinical and family care, we should be more concerned with two important factors to PTSD: income and cancer stage.

The present results indicate that social support significantly and negatively correlates with PTSD symptoms in patients with ovarian cancer in China (P < 0.01), in accordance with prior studies. Jacobsen [70] found that lower social support predicts greater PTSD symptoms severity. In other words, higher levels of PTSD symptoms are associated with less social support [71]. In recent studies, Yang [65] showed that social support from one’s family is significantly associated with PTSD symptoms in Chinese individuals with bladder and kidney cancers. Liu [64] demonstrated that PTSD symptoms are negatively associated with social support in patients with hematological malignancies in China. Wang [72] also confirmed that social support is negatively associated with PTSD symptoms in Chinese patients with central system tumors.

In addition, our results show that hope and resilience correlated negatively with PTSD symptoms, concordant with prior results [73] despite different samples. However, among Chinese patients with hematological malignancies, hope and resilience have not shown significant negative associations with PTSD symptoms [64]. In contrast, Besser [74] showed that resilience negatively associated with acute PTSD symptoms. Resilience is inversely correlated with self-reported PTSD symptoms in most survivors of critical illness [75]. Another study found that nurses with PTSD symptoms had significantly lower total resilience scores compared to those without PTSD symptoms [76]. Wright [77] reported that kidnapping victims without PTSD symptoms reported higher individual resilience. Joscelyne [78] found that responders with high levels of resilience reported minimal symptoms of PTSD symptoms. In general, to our knowledge, relevant studies remain limited. Therefore, we provided reliable evidence of the association between hope, resilience, and PTSD symptoms in Chinese patients with ovarian cancer.

Moreover, the results of the bootstrapping test indicate that hope and resilience act as mediators in the association between social support and PTSD symptoms in Chinese patients with ovarian cancer. The mediatorstested in our study differs from most previous studies [72, 79–81], in which authors have mostly chosen self-efficacy [72], emotional abuse [79], sleep disturbances [80], and negative perception of social support [81]. Although Li [73] also suggested that resilience partially mediated the relation between psychological stress with depressive and anxiety symptoms, their research differed from ours in terms of population, dependent and independent variables, and even the resilience scale to some extent. Although we all used the Resilience Scale-14, the original scale of the Chinese revision we used is from Block J [59]. We used a4-point Likert scale, while Li [73] used a 7-point Likert scale. Both studies, however, showed that resilience is a significant negative mediator.

It is noteworthy that the proportion of the mediating effect of hope for social support (43.37%) was higher than the proportion of the mediating effect of resilience (10.64%). This might be because of potentially different features of hope and resilience. Hope is a multidimensional dynamic life force. Hope is characterized with confident and uncertain expectation of achieving a good future. If the individuals are hopeful, they will be confident that things will turn out to be fine. Then they are more likely to dedicate themselves to meaningful work, for example, participating in the social network; committing to family relationships; relieving stress and tension; and keeping optimistic attitude toward life. Hence, hopeprobably plays a relatively more important role in tapering off the PTSD symptoms. Resilience represents a homeostatic rebounding to a prior level of functioning after a stressor or trauma. If a patient is high in resilience, she will handle or rebound from adversity successfully. Although the mediating effect of resilience is not as high as hope for social support, we should not ignore it. Inter-correction between hope and resilience may occur. An individual who is more resilient or, who has gone through a resilient recovery, probably may be more hopeful. In addition, an individual, who has seen the hope of the future, may be more likely to resume their normal pre-stressor level of functioning. A strong correlation between resilience and hope has been indicated among advanced cancer patients (0.63; p < 0.05)[82] and middle aged adults (0.59;p < 0.05)[83].

In the present study, social support explained 14.7% of the variance in PTSD symptoms, while hope and resilience together explained 17.0% of the variance in PTSD symptoms. It seems that intrapersonal psychological resources (hope and resilience) are more important than perceived interpersonal resources (social support), or at least that they are about equal. Future studies should examine the effect of these different kinds of psychological resources on PTSD symptoms.

Finally, it is necessary to pay more attention to and invest more resources into not only social support, but also hope and resilience concerning the prevention of PTSD symptoms among patients with ovarian cancer. Since social support correlates with PTSD symptoms negatively, we should pay enough emphasis on the assessment for deficits in social support in ovarian cancer patients. In addition, clinical doctors and researchers should be aware that hope and resilience might play a role in the outcomes among cancer patients. For example, Fava GA developed psychotherapeutic strategy to increase psychological well-being and resilience in mood and anxiety disorders [84].

Some limitations should be mentioned. First, the present study was a cross-sectional study. Accordingly, we could not draw important conclusions regarding the trends of the relation among social support, hope, resilience, and PTSD symptoms by time. Thus, causality of each variable could not be determined. However, there might be reverse causality. For example, high resilience can possibly promote the social support [85]. An evidence has confirmed that greater resilience enables individuals maintain better social networks and seek new social support resources, and own higher perceived social support; Moreover, high-hope individuals tend to be social beings, they are good at interpersonal interactions and prefer to explore social support [86]. Furthermore, to individuals with PTSD symptoms, it is possible that they will feel inferior, autistic, and refuse to mention their misfortune story to others and even just explore social support. For individuals with PTSD, the acceptance of social support may be particularly difficult because of core symptoms such as avoidance, alienation, detachment and emotional numbing[32].

Second, we used convenience sampling, not stratified sampling, and we only recruited patients with ovarian cancer from two hospitals of China Medical University. Third, we only focused on some psychological resources; other factors such as self-efficacy, optimism, and self-esteem needed further consideration. Despite these limitations, we assessed the mediation with multiple mediators (hope and resilience) in the association between social support and PTSD symptoms in patients with ovarian cancer in China.

In the future, we will make further research: to use longitudinal designing methods to infer causality; to consider the relation between self-efficacy, self-esteem and optimism with PTSD symptoms; to recruit ovarian patients from the south and west regions in China.

## Conclusions

This study provides a self-report prevalence of PTSD symptoms among Chinese patients with ovarian cancer. After controlling demographic and clinical characteristics, we demonstrated multiple mediation roles of hope and resilience in the association between social support and PTSD symptoms. Social support correlated negatively with PTSD symptoms. Social support explained 14.7% of the variance in PTSD symptoms. When hope and resilience were added, we found they together explained 17.0% of the variance in PTSD symptoms. The findings indicated that hope and resilience partly mediated the correlation between social support and PTSD symptoms. Patients with higher levels of hope and resilience might show a strengthened effect of social support on PTSD symptoms. Interventions to relieve PTSD symptoms should be focused on not only social support, but also hope and resilience.

## Supporting information

S1 Data Supporting dataset. The supporting dataset includes the data underlying our findings in this study.
(XLSX)
